# Supplementary material for: Immunogenicity and Protective Efficacy of Dose-Sparing Epigraph Vaccine against H3 Swine Influenza A Virus
Source: Vaccines (Basel). 2024 Aug 22;12(8):943. doi: 10.3390/vaccines12080943 (PMC11359338; doi:10.3390/vaccines12080943)
Supplement: Supplementary file 1 [file vaccines-12-00943-s001.zip › vaccines-3150794-supplementary.pdf]

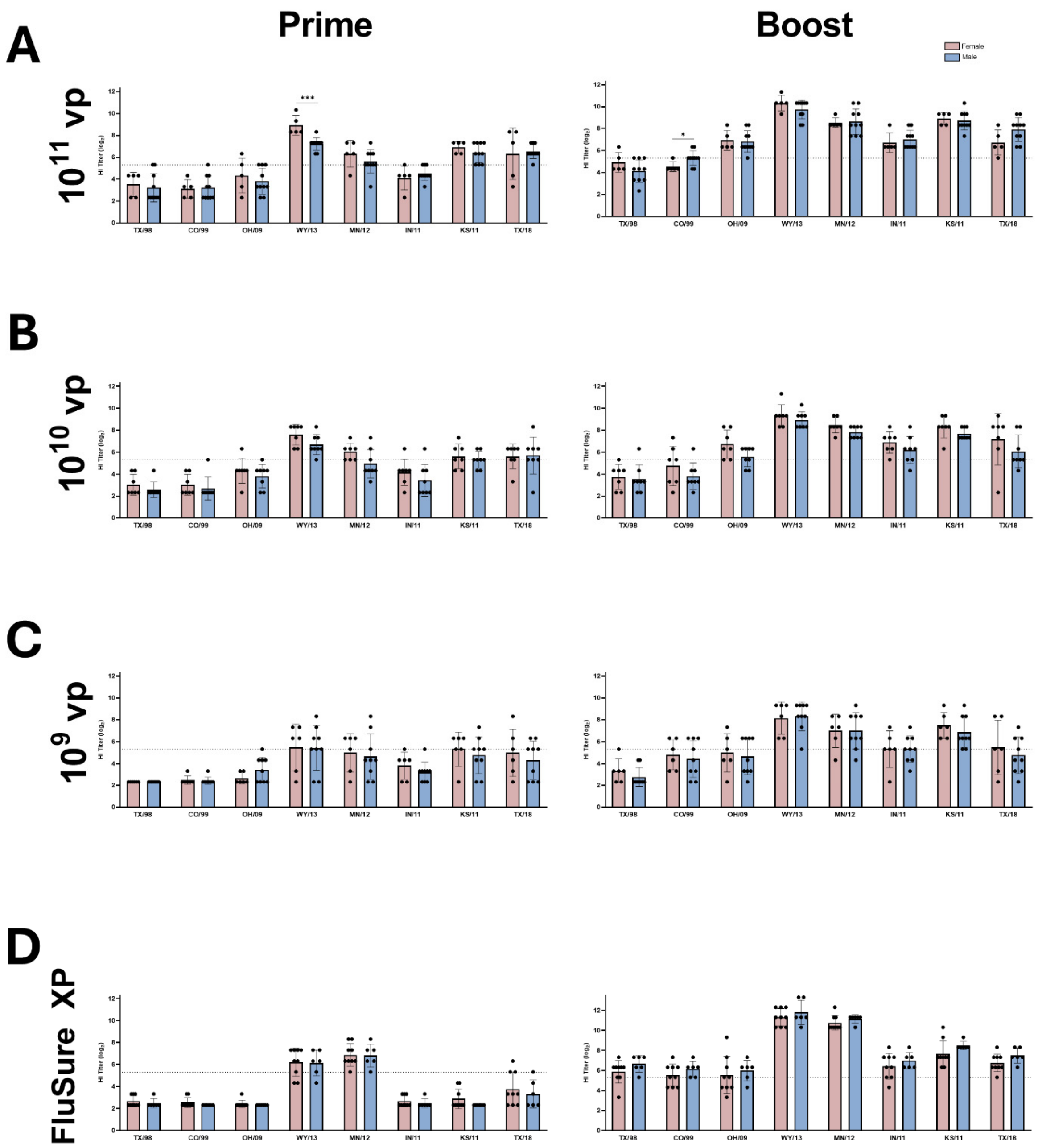

Supplemental Figure S1. Sex-based differences in antibody responses. Male- and female-specific hemagglutination inhibition (HI) antibody responses were assessed for each dose after a prime and boost immunization. Pigs were vaccinated with Epigraph at (A) 10^{11}vp (*n* = 5 females, 10 males), (B) 10^{10}vp (*n*= 7 females, 8 males), (C) 10^{9}vp (*n* = 6 females, 9 males), or (D) FluSure XP (*n* = 9 females, 6 males). Data is presented as the mean ± SEM. Statistical analysis was done by an unpaired *t*-test and an α value of 0.05 was considered statistically significant; *** $p < 0.001$.

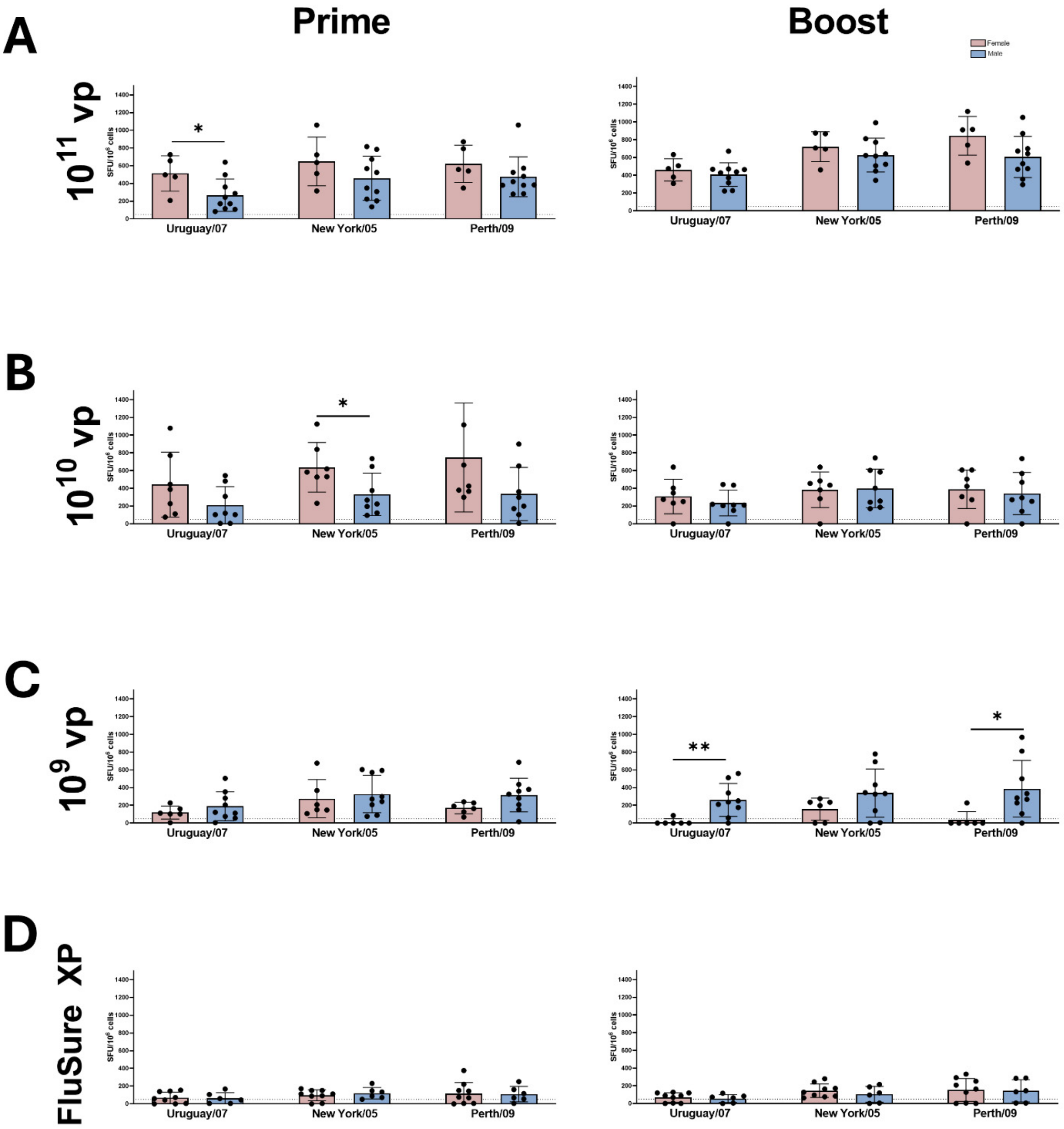

Supplemental Figure S2. Sex-based differences in T cell responses. Male- and female-specific T cell responses were assessed by IFN-γ ELISpot for each dose after a prime and boost immunization against Uruguay/07, New York/05 and Perth/09. Pigs were vaccinated with Epigraph at (A) 10^{11}vp (n = 5 females, 10 males), (B) 10^{10}vp (n= 7 females, 8 males), (C) 10^{9}vp (n = 6 females, 9 males), or (D) FluSure XP (n = 9 females, 6 males). Data is presented as the mean ± SEM. Statistical analysis was done by an unpaired t-test and an α value of 0.05 was considered statistically significant; * $p < 0.05$, ** $p < 0.01$.

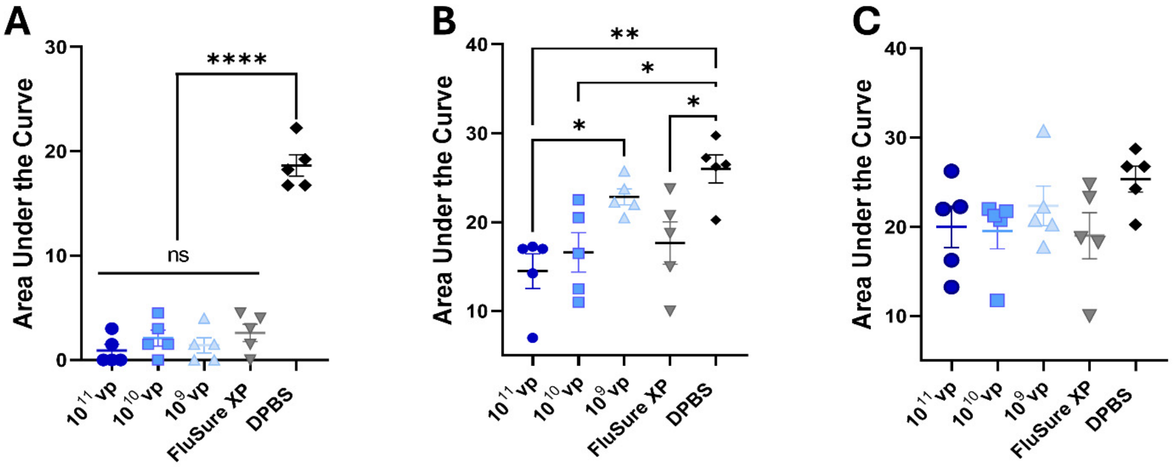

Supplemental Figure S3. Area under the curve (AUC) plots for daily nasal swabs. Total area under the curve (AUC) of infectious virus present in nasal swabs collected from each pig over the course of infection was calculated and plotted after challenge with (A) A/swine/Ohio/11SW87/2011, (B) A/swine/Texas/4199-2/1998, or (C) A/swine/Texas/ A01785781/2018. Data are presented as the mean ± SEM. Statistical analysis was compared between groups using a one-way ANOVA with Tukey's multiple comparisons follow up test; ns- not significant, * $p < 0.05$, ** $p < 0.01$, **** $p < 0.0001$.
